# Use of conjoint analysis to weight biosecurity practices on pasture-based dairy farms to develop a novel audit tool—BioscoreDairy

**DOI:** 10.3389/fvets.2024.1462783

**Published:** 2024-12-10

**Authors:** Siobhan M. O Donovan, Conor G. McAloon, Luke O'Grady, Timothy Geraghty, Alison Burrell, Marie-Claire McCarthy, John Donlon, Jamie A. Tratalos, John F. Mee

**Affiliations:** ^1^Moorepark, Teagasc, Animal and Grassland Research and Innovation Centre, Fermoy, Co. Cork, Ireland; ^2^School of Veterinary Medicine, University College Dublin, Dublin, Ireland; ^3^Centre for Veterinary Epidemiology and Risk Analysis, UCD School of Veterinary Medicine, University College Dublin, Dublin, Ireland; ^4^SRUC Veterinary and Analytical Services, Pentland Science Park, Edinburgh, United Kingdom; ^5^Animal Health Ireland, Carrick-on-Shannon, Co. Leitrim, Ireland; ^6^Department of Agriculture, Food and the Marine, Cork, Ireland; ^7^Animal and Bioscience Research Department, Teagasc, Animal and Grassland Research and Innovation Centre, Dunsany, Co. Meath, Ireland

**Keywords:** biosecurity, risk assessment tool, questionnaire, best-worst scaling, scores, weightings, dairy, cattle

## Abstract

Risk assessments are important tools to identify deficits in biosecurity management practices. A major strength of some existing tools is that they facilitate cross-country comparisons. However, a weakness is their failure to account for unique intra-national farming enterprise structures such as, for example, pasture-based dairying. Currently, there are no suitable biosecurity risk assessment tools applicable to pasture-based dairying as practiced in Ireland. In addition to a need for enterprise-specific biosecurity risk assessment tools, the weighting of risk scores generated by these tools needs to be context-specific to ensure validity in assessing biosecurity risks in the farming sector of interest. Furthermore, existing biosecurity audits rely exclusively on respondent recall to answer questions about management practices. To address each of these limitations of existing biosecurity risk assessment tools we developed and optimised a new biosecurity risk assessment tool (BioscoreDairy) designed to assess the biosecurity status of pasture-based dairy farms in Ireland. It consists of two parts, a biosecurity questionnaire and a cattle movement records audit. A questionnaire was developed on biosecurity management practices appropriate for a pasture-based dairy system. Multiple national expert groups were leveraged to provide weightings for the different management practices in the questionnaire using the best-worst scaling methodology of MaxDiff. The results of this process provided a numerical categorisation that could then be used to assign scores to the individual biosecurity management practices. These practices were grouped into three biosecurity areas; risk of disease entry, speed of disease spread and diagnosis of infection. Within each of these three areas, a traffic light system was used to compare a farm’s biosecurity risks to other similar farms—least risk (green; within the top third of farms), concerning practice (amber; middle third) and worst practice or greatest risk (red; lowest third). In addition to these scores, the cattle introduction profile of a herd over the previous 3 years, based on nationally recorded data, was audited, compared amongst dairy farm enterprise subtypes, and included in the BioscoreDairy report. BioscoreDairy is therefore the first biosecurity risk assessment tool tailored to pasture-based dairy farm systems, both for individual farm reporting and for benchmarking against comparable farms.

## 1 Introduction

Biosecurity is defined by the World Organisation of Animal Health as “*a set of management and physical measures designed to reduce the introduction (bioexclusion), establishment and spread (biocontainment) of animal diseases, infections or infestations to, from or within an animal population*” (1). To identify biosecurity deficits in farm practices and compare such practices with similar farms, various biosecurity audit tools have been developed. Such assessments are usually based on a questionnaire designed for use by farmers (self-assessment) or, more commonly, by professional service providers (external assessment). These questionnaires may be pathogen/disease-specific, e.g., Johne’s disease (2) or they may be pathogen/disease-agnostic, e.g., (3). In the intensive animal production sectors, there is evidence of the beneficial impact of implementation of biosecurity measures on antimicrobial use and on production indices (4).

As biosecurity measures are adopted to a lesser degree on cattle farms compared to other more intensive enterprise types (5), the need for comprehensive biosecurity auditing of cattle farms is possibly even greater. Additionally, some of the lack of adoption of biosecurity measures on cattle farms may be explained by the limited suite of robust biosecurity audit tools currently available to assess, and hence facilitate benchmarking of biosecurity performance. This is important given that previous research has demonstrated that benchmarking motivates farmers to improve management practices (6).

Audit tools that have been published to date are generally developed to address biosecurity concerns within a particular farming sector, e.g., pig farming, or within a specific country [e.g., (2)]. An exception to this is the Biocheck, a robust suite of questionnaires which have been developed in Belgium but are used internationally for multiple enterprises; pigs (7), poultry (8), dairy (9) and beef (10). A major advantage of these tools is that they facilitate cross-country comparisons and benchmarking. However, within each farming sector, there may be significant variation in how farms are managed and even the epidemiology of livestock infectious diseases between countries. This is arguably greatest in the dairy sector (compared to pig or poultry sectors), which displays variations of seasonal vs. non-seasonal calving, confinement vs. pasture-based systems, both within- and between countries. It is therefore possible that more generic tools, which aim to facilitate greater ‘external’ comparisons between countries, may be less applicable and/or valid internally. Accordingly, McCarthy et al. (11) assessed publicly available dairy cattle biosecurity questionnaires internationally and concluded that none adequately suited pasture-based dairy farming, such as that practiced in Ireland.

Audit tools should weight biosecurity deficits according to their perceived risk with respect to farm biosecurity. However, it is likely that the weighting and prioritisation of biosecurity practices for one country may differ to that of another. In addition, terminology and language concerning different practices is likely to differ between countries, so some local ‘translation’ is likely to result in more accurate data collection regarding biosecurity practices. This process is particularly important if the tool is to be used by farmers rather than, for example, being collected by a veterinarian. Finally, a further potential weakness of existing tools is their limited ability to gather accurate data on cattle movements into the farm, when they rely on farm recall. Cattle introductions are the most important risk factor for introduction of infectious pathogens into a herd (12). As with all answers in a questionnaire, there is a risk of gathering inaccurate information due to recall, recency or other responder cognitive biases (13). While this may not be critical for some information (e.g., whether milk recordings are carried out or not), it is essential that cattle introductions are accurately documented when assigning a biosecurity risk status to a farm. There is therefore a need to collate accurate/objective data on cattle introductions with other farmer biosecurity behaviours to get a more complete perspective on the farm’s biosecurity status.

In Ireland the requirement for a robust, holistic biosecurity audit tool has become more important with the recent major demographic changes in the dairy industry. Irish dairy farming is based predominantly on small herds (mean 90 cows) which are seasonal calving, pasture-based, and family-run (14). Nationally, there is high regional density of dairy cattle, high inter-farm cattle movements, with some infectious endemic diseases under legislative control (e.g., bovine tuberculosis, bovine brucellosis, bovine viral diarrhoea), others under voluntary control (e.g., Johne’s disease, mastitis/SCC, infectious bovine rhinotracheitis) and many others with no recognised national control programme (e.g., leptospirosis, salmonellosis, cryptosporidiosis) (15),. Since the European Union milk production quota was abolished in 2015, the Irish national dairy herd has expanded significantly (16). This expansion highlighted the need to address biosecurity risks from increased cattle movements. Hence the National Farmed Animal Biosecurity Strategy (NFABS 2021–2024) was introduced in 2021 (17). This strategy was designed to place increased emphasis on prevention of disease entry and spread within a herd.

To deliver the national biosecurity strategy a context-specific biosecurity audit tool tailored to pasture-based dairy production is needed. Therefore, the objective of this study was to develop a farmer-facing biosecurity scoring audit tool for use on pasture-based dairy farms, based on an expert-weighted risk assessment (RA) questionnaire and cattle movement data to capture and benchmark dairy farms.

## 2 Materials and methods

### 2.1 Risk assessment (RA) questionnaire development

A novel biosecurity RA questionnaire was developed using participatory design methodology. The aim was to produce a farmer-facing questionnaire that would assess farm biosecurity-related behaviours and performance generically (i.e., not disease-specific) across multiple infectious diseases and create national benchmarks. Unlike existing tools, the overall design was not predetermined by the bioexclusion and biocontainment dichotomy. The questionnaire for this new biosecurity tool was initially based on McCarthy et al. (11). Questions from this survey were cross checked with two existing publicly available biosecurity questionnaires: Biocheck (18), and the Irish Johne’s Control Program (IJCP) Veterinary Risk Assessment and Management Practices (VRAMP) tool (19), to ensure no management practices were overlooked. The full list of questions were compiled and reviewed to find duplicate questions, which were excluded.

The consolidated list of questions were reviewed by the Animal Health Ireland (AHI) Biosecurity Technical Working Group (TWG).^1^ The backgrounds of the membership of this group comprised veterinary practitioner, university veterinarian, pharmaceutical company veterinarian, AHI veterinarian, Department of Agriculture, Food and the Marine veterinarian, research veterinarian, dairy specialist agricultural adviser, sire performance centre manager and health psychologist, To aid with this technical review, an evaluation document with three key guidelines was circulated to members. This document specified that each reviewer should independently review the questions with a focus on three key criteria: (1) alternative information source—could this information (or a reasonable alternative) be provided automatically from the national Animal Introduction Movement (AIM) database maintained by the Department of Agriculture Food and Marine. AIM is the central data base of Ireland for cattle, pigs, sheep and goats (20) (yes/no), (2) redundancy—could this question be removed or amalgamated with another question in a way that would not lead to significant loss of vital information, and (3) practicality—is this a practice that could reasonably be modified on a commercial (dairy) farm? Following this review, the questionnaire was revised by two of the authors (JFM and CMA) based on the feedback gained from the technical review process.

Next, the biosecurity questionnaire was reviewed in full by a panel consisting of two of the authors (CMA and JFM), two dairy farm advisors, and a Department of Agriculture Food and the Marine (DAFM) veterinarian, to find consensus on the language used in the document with a particular emphasis on language that would be familiar to a dairy farmer, without compromising the specific information the question aimed to collect. Finally, the questionnaire was then sent to five dairy farmers (four male, one female; farm managers of dairy research herds) who were asked to complete it and to provide additional feedback on the technical content, the language and the format.

The farmers’ comments were used to revise the questionnaire again by members of the project team (CMA, JFM, LOG, TG). The survey was then divided into 4 sections: risk of disease entry, speed of disease spread within herd, diagnosis of infection and baseline resilience/vaccination. Next, the questionnaire was reviewed by a chartered health psychologist from Animal Health Ireland (AB) to identify any language which may influence responses, and to identify any weaknesses within the questionnaire’s clarity.

Irrespective of the enterprise a biosecurity questionnaire is designed for, the language used and the responders’ perception of the meaning of questions may influence the answers provided. One way of reducing this bias is to conduct cognitive interviews (CI) with pilot respondents. Cognitive interviews are conducted in order to evaluate individuals’ understanding of the survey through “think aloud” protocols and verbal probes (21). They are a qualitative development method used to aid the development of the survey by helping the design team to investigate the clarity of the survey and gain the responders perception of each question. They also highlight whether the survey achieves the overall objective (22). Thus, five cognitive interviews (CI) were carried out by the first author (SOD) to get feedback from dairy farmers. These interviews were focused on identifying questions that were unclear and resulted in confusion for the farmer. The CIs were carried out either in-person or online via video call. The farmers were provided with a copy of the revised questionnaire to which they had to respond orally, reading the question aloud and choosing their answer. Where hesitation or a reaction to the question and/or answer was observed, they were asked what caused such reaction or confusion. From this process any questions which caused confusion or misunderstanding were highlighted. So too were answer options which may have been omitted. Finally, all cognitive interviews were reviewed and from there the questionnaire was edited again.

The edited document was finally reviewed by 6 project members (SOD, JFM, CMA, LOG, TG, AB). The questionnaire (*n* = 75 questions) was then uploaded to an online survey software platform—Survey Monkey (23).

### 2.2 Risk assessment questionnaire scores and weightings

Scores were generated separately for each of the three sections of the questionnaire [(1) risk of disease entry (2) speed of disease spread and (3) diagnosis of infection] which in turn had to be generated from individual question scores. Section 4 of the questionnaire relating to herd resilience and vaccination was not scored as this section contained questions (e.g., “are health traits within the EBI sub-indices part of selection criteria when breeding animals?”) of qualitative or disease-specific value only, as such actions were not deemed to directly affect the general risk of disease entry or spread.

#### 2.2.1 Within-section scores

Scores were assigned to each question within each of the three sections, based on its perceived risk to farm biosecurity. Scores were derived using a best-worst scaling (BWS) approach (24). This method collects paired comparison data, therefore forcing the expert to make compromises in their decisions (25). Using this approach, experts were provided with sets of four management practices at a time relating to one of the three sections and asked to identify the best (lowest risk for biosecurity) or worst (largest risk for biosecurity) practice relating to the biosecurity area (e.g., risk of disease entry). Repeating this process multiple times (12–16 depending on the number of questions per section) with multiple combinations of options, and across multiple users, facilitates the estimation of relative weights for each of the individual responses.

All possible questionnaire responses (attributes) for each question were transformed into statement format for each of the three sections: risk of disease entry: 101 attributes, speed of disease spread: 96 attributes and diagnosis of infection: 22 attributes. Random subsets of four statements were presented each time, along with a question relating to which would have the best (or least detrimental) or worst (or most detrimental) impact on the aspect of biosecurity covered in that section of the questionnaire. The presentation of subsets was repeated multiple times for each respondent to allow accurate ranking of responses.

Scoring was conducted by representatives (*n* = 39) of five preselected veterinary groupings; the research project team (*n* = 5; university veterinarian, research veterinarian, diagnostic laboratory veterinarian, postgraduate PhD student), the Animal Health Ireland (AHI) biosecurity Technical Working Group (TWG) (*n* = 8; see membership detail above), Irish diplomats of the European College of Bovine Health Management (*n* = 7; private veterinary practitioner, Department of Agriculture, Food and the Marine veterinarian, university veterinarian), biosecurity specialists in Department of Agriculture Food and Marine (DAFM) (*n* = 14; veterinary biosecurity officer, veterinary pathologist, veterinary epidemiologist, veterinary inspector, veterinary research microbiologist) and Irish private veterinary practitioners (*n* = 5). Conjointly software (26) was used to gather and analyse the responses from best-worst scaling. Three webinars were hosted in which the questionnaire and the BWS technique were explained, and training was given on how to answer a BWS survey using a demo scenario. The 39 experts were then asked to complete a separate BWS survey for each of the three sections individually and reminded that their responses were to be agnostic of any single pathogen or disease. All responses were anonymous.

Settings were applied to the BWS system so that the format at which the attributes (statements) would appear, how many times they would appear, and recommended time taken to complete the exercise could all be altered. Therefore, when applying settings to the BWS, the attributes (statements based on farm practices) shown per set at any time were automatic. This meant that the number of times an attribute could appear throughout the BWS was controlled and all attributes appeared an equal number of times. The order at which attributes appeared was randomised, so no two participants received the same set of attributes at any time. Analytical settings were also applied. A confidence interval of 90% was set. A setting to eliminate low quality responses was applied, with warnings appearing on screen when a participant was completing an exercise too fast; this response was deemed low quality. Where this warning appeared, it was suggested that the participant was not giving their honest opinion or full concentration. The process of scoring was carried out within the 3 sections rather than across all sections. This process was repeated for all the statements/attributes in each of the three sections.

**Figure 1:**
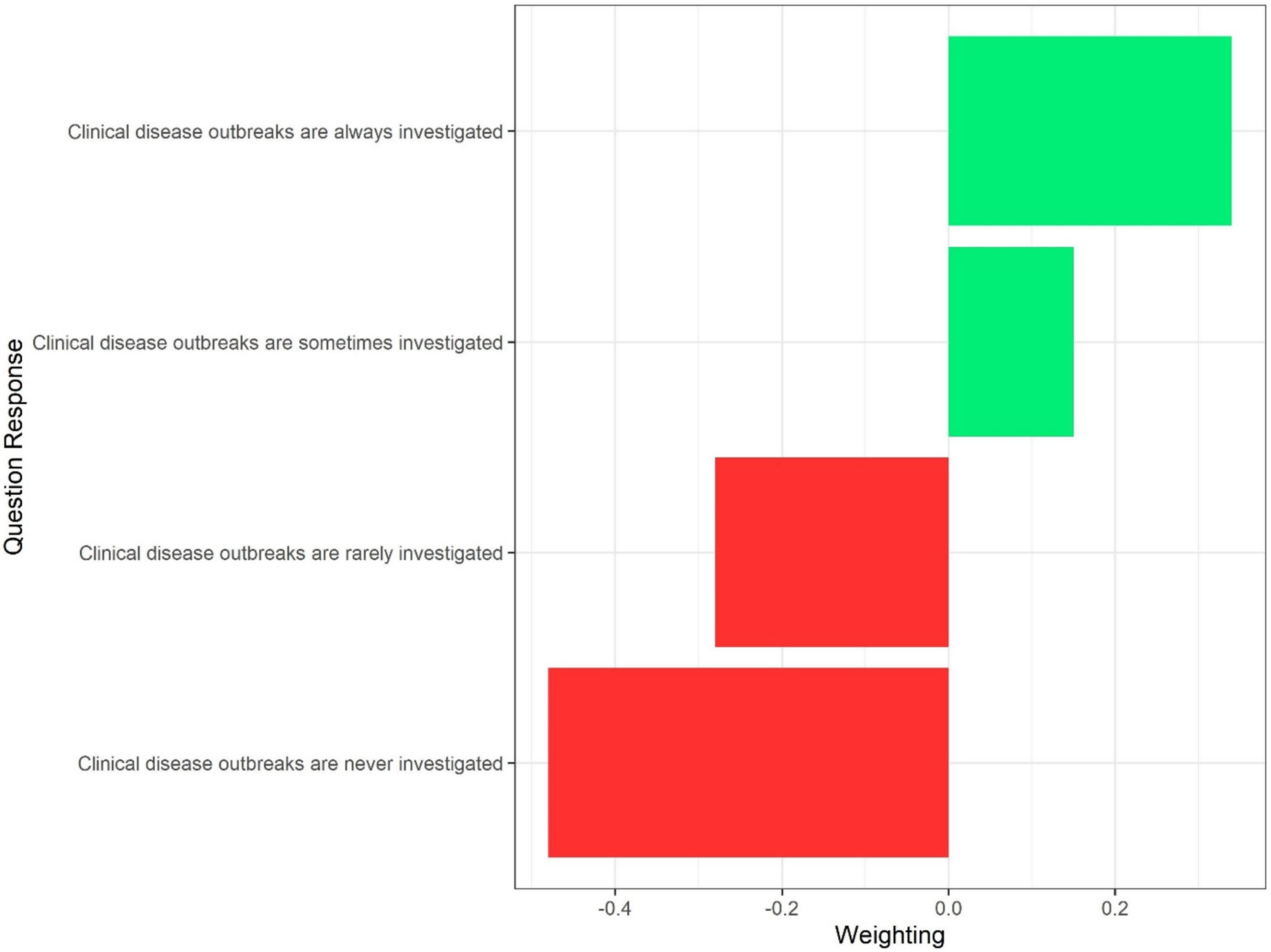
Example of best worst scaling weightings for a single question on investigation of a clinical disease outbreak.

The Best Worst Scaling theory, using Maximum—Difference method, allocates values to each of the answer options per question (within each section), thus allocating a score to each answer option (Figure 1). These BWS scores were then reviewed in detail by the authors to identify cases where non-biologically plausible weights had been assigned, that is, weights that placed the ranking of responses in an order which conflicted with biological plausibility. When this occurred, one of four options was followed: When a single biologically higher risk response was assigned a lower risk score than the next response, but the authors believed, on discussion, that the risk was similar from both, both responses were assigned the same weighting, which was calculated as the mean of the weights for the two responses. In contrast, when the authors believed there was a clinically-relevant difference between the risk, for example, if the highest or lowest risk response (from a biological perspective) was assigned a score placing it out of order with the rest of the options, this option would be assigned a value equal to the highest or lowest weighted response. If multiple responses within the question appeared to have biologically inappropriate weightings (relative to the other attributes within that question), but the authors believed these differences to be minor, individual attributes were assigned a weighting of zero and the attribute did not contribute to the overall risk score. Finally, if multiple responses within the question appeared to have biologically inappropriate weightings, and the authors deemed these risks significant, weightings were re-assigned according to the biologically plausible ordering of the risks.

To obtain the total score per section, responses for each survey question were matched to the corresponding BWS weighting and these weightings were added together across all the questions for that section. Then the BWS weighting total per section was expressed as a percentage of the maximum possible score from all questions in that section.

### 2.3 Cattle introduction tool

Cattle introduction indicators were developed for each farm: in degree, inward strength and secondary inward degree. In degree was defined as the number of cattle moved onto the farm in the previous 3 years, inward strength was defined as the number of herds from which cattle were introduced onto the farm in the previous 3 years, and secondary inward degree extended this to the herds from which source herds introduced cattle from. Herds were categorised using the cattle enterprise classification system recently developed by Brock et al. (27) and benchmarked to other herds within this category according to the median and 10th and 90th percentiles and the overall distribution. Data to compute these metrics reside within the Animal Identification and Movements (AIM) database. Therefore, this section of the score can be populated based on routinely collected data alone and allows each herd to be compared against all herds in the country.

### 2.4 Farm biosecurity report

Finally, the scores from the biosecurity questionnaire and the cattle introduction tool data were combined in a farm biosecurity report called BioscoreDairy. The automatic generation of the report was coded using R (28). The coding process formulated the farm report by linking the farmer’s responses from the questionnaire and the BWS weightings. Farm scores were benchmarked against the records of all other farms that have taken the assessment. For illustrative purposes, the 33rd and 67th percentiles of the distribution of scores for each section were calculated and a visual plot created to summarise the farmer’s score, colour-coded according to their position in the distribution compared to other similar farms (low, medium, high). Farms’ sections (e.g., disease diagnosis) with the best scores (lowest risk) one third were coded green, those in the bottom one third were coded red, and those in the middle third are coded amber.

## 3 Results

### 3.1 Biosecurity questionnaire and weightings

The final questionnaire is provided in Appendix 1. It consisted of 70 questions across four sections (section one risk of disease entry: *n* = 28; section two speed of disease spread: *n* = 21; section three diagnosis of infection: *n* = 12; section four baseline resilience/vaccination: *n* = 9). The questionnaire took approximately 16 min to complete. This was calculated using the survey platform where the time was recorded from when the link was opened to the time of submission. A sum of all timings was calculated and divided by the number of responses to obtain an average.

The weightings assigned to each biosecurity practice in the three sections subjected to the weighting process (risk of disease entry, speed of disease spread and diagnosis of infection) are shown in Supplementary Tables S1–S3.

### 3.2 Cattle introduction tool

**Figure 2:**
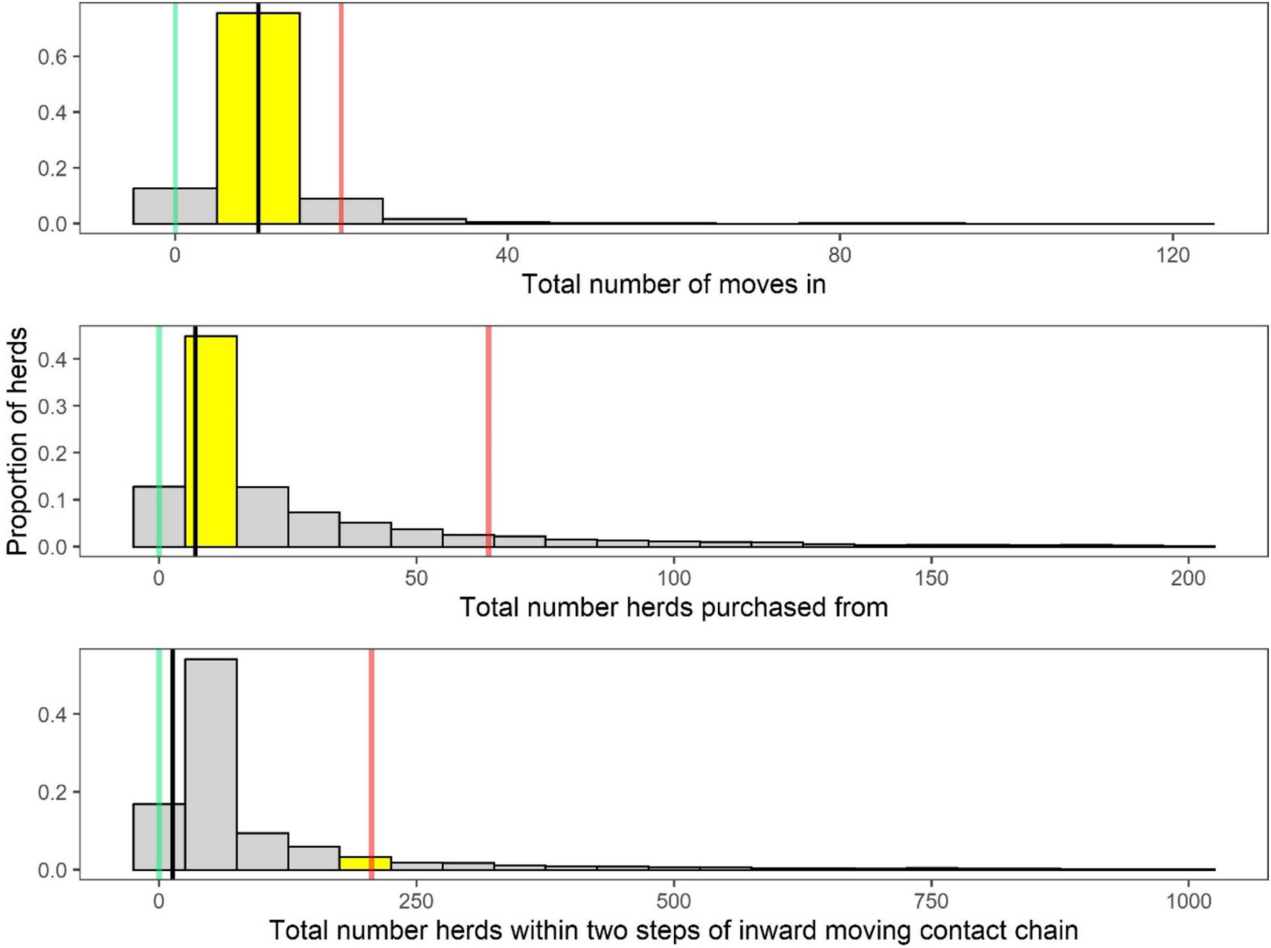
An example of an animal introduction summary figure from a BioscoreDairy report. Animal introductions are based on the number of animals bought in (first frame) and the number of source herds (frames two and three). For each farm, these metrics are compared to 50 other similar comparable herds in the same herd category [(27) classification system). The distribution of these data for the comparator herds are shown as grey bars in the three frames. The position of the individual example herd in each graph frame is indicated by the yellow bar. The green line represents the position of the lowest-risk (10th percentile) herds, the black line indicates the position of the average herd, and the red line indicates the position of the highest-risk (90th percentile) herds, nationally.

The number of cattle introductions and number of source herds were analysed for each individual herd and compared with data from comparable herds in the national cattle movement database. Figure 2 shows a plot for an example herd: the red line represents the highest risk (90th percentile), the black line represents the median herd, while the green line represents the lowest risk (10th percentile) position of herds nationally. The position of the example herd is highlighted in yellow.

### 3.3 Farm biosecurity report (BioscoreDairy)

**Figure 3:**
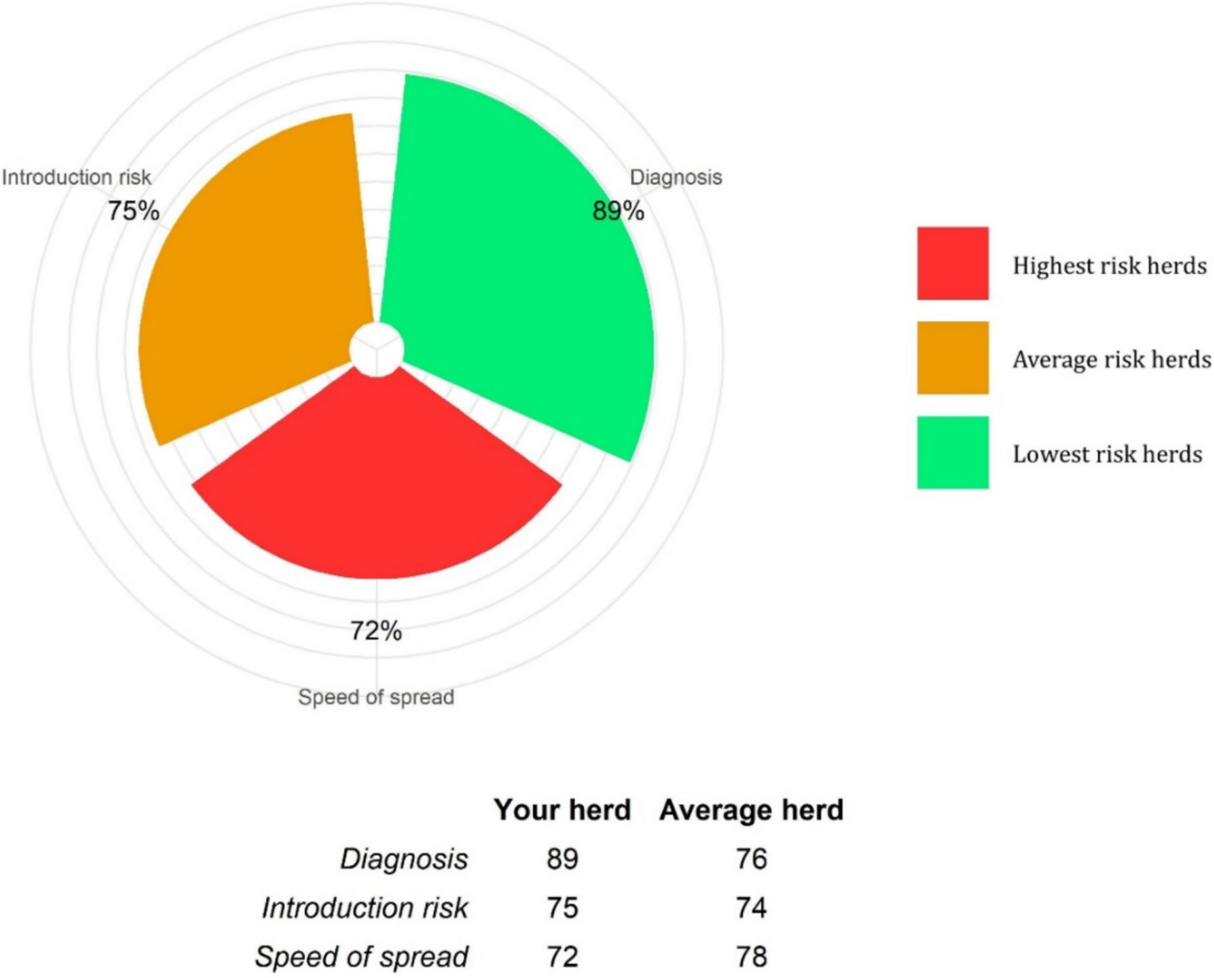
An example of a biosecurity score summary, from a farm BioscoreDairy report. The example farm score percentages for disease diagnosis, infection introduction risk and speed of infection spread risk, are a percentage of the maximum score percentage possible for each of these sections. These example farm score percentages are benchmarked against other comparable herds in the BioscoreDairy database. Higher score percentages indicate lower risk. The distribution of score percentages for the comparator herds are colour-coded into low risk [<33rd percentile (green); average risk between the 33rd and 67th percentile (amber); and high risk >67th percentile (red)].

An example of a farm biosecurity report is provided as Supplementary material 2. The final report summarised the three section scores and categorised the risk of disease entry, speed of disease spread and diagnosis of infection, each separately as low, moderate or high, according to whether their scores were above or below the 33rd or 67th percentiles, respectively (Figure 3). Risk of disease entry was subcategorised, and scored, into the number of cattle introduced and the sources of these cattle and the farmer’s responses to the questionnaire. Speed of disease spread was subcategorised, and scored, as that between sick and healthy cattle, adult to young (pre-weaned calves, weaned calves, yearlings) cattle, young to young cattle and adult to adult cattle, to give an overall farm speed of disease spread score. Answers regarding herd resilience and vaccination were not scored; however, they were recorded for four cattle age categories; pre-weaned calves, weaned calves, yearlings and adults.

## 4 Discussion

This study represents, to our knowledge, the first development of a biosecurity risk assessment tool for pasture-based dairy farms. It makes use of electronic movement data regularly collected in many countries. The tool has been optimised for Irish dairying based on language and phrasing as well as local expert weighting but can easily be adapted as appropriate for a particular country with similar production systems.

Our study is not unique in seeking to develop methods to collect management practices relevant to herd biosecurity. To date, similar studies fall into three broad categories: 1. Questionnaires with a broad biosecurity focus, developed primarily for research use, to capture data relevant to a specific research project or study (29); 2. Questionnaires with a broad biosecurity focus developed for practical or commercial use, for example Biocheck (18) and 3. Questionnaires developed for a specific disease, developed for practical or commercial use, for example the paratuberculosis Risk Assessment (30).

While the underlying data our study seeks to capture are similar to those in tools developed in each of these examples, there are important differences. Firstly, in contrast to comparable studies, a particular focus of this study was to create a tool which was designed to be ‘farmer-facing’, i.e., for the farmer to complete, rather than an animal health professional. Therefore, we adopted a multidisciplinary approach to questionnaire development including biosecurity experts, social scientists, dairy farmers, dairy advisors, and veterinarians. In this way, question selection and wording were refined through a robust iterative process which including multiple iterations with dairy farmers to ensure language was appropriate, and to reduce any potential for misunderstanding.

Secondly, whilst the weighting and score allocation approach taken in this study can be compared to other scoring systems developed for biosecurity, in non-research settings, two differences in particular are worth noting. Firstly, a strength of the BioscoreDairy approach is the steps taken to weight the responses of farmers according to the level of risk assigned, with particular focus on management practices. The application of BWS is rarely applied to the domain of biosecurity and is a time-efficient approach to assigning objective rather than subjective scores from a large network of experts. The BWS allows for a multitude of expert opinions to be compiled into weights and for these to be converted into percentages or scores. Secondly, whilst the system we developed is developed specifically for pasture-based dairying and therefore useful for dairy farms internationally with similar production methods, the weighting applied to this scoring system is, we believe, context-specific. Our study has outlined how the series of steps taken to tailor the system to the Irish system may act as a robust framework for the development of and refinement of other biosecurity assessments.

Finally, whilst existing disease-specific risk assessments are commonly used in Ireland, our study addresses a particular challenge with these approaches. For example, the Johne’s disease Risk Assessment is a widely adopted component of international Johne’s disease control programmes (31). However, based on qualitative research of Irish farmers, recent work from our group has argued for integrated disease preventive strategies into group programmes in so far as possible, as opposed to disease-specific programmes (32). The tool developed in this study represents a means by which multiple diseases can be mitigated in an integrated way, as opposed to a disease-specific manner, potentially improving farmer engagement.

Following the development of BioscoreDairy, it is now being utilised on a cohort of Irish dairy farms to both collect biosecurity data and to evaluate its suitability for field use in the Irish NFABS. BioscoreDairy could be used in other countries which operate a pasture-based dairy system such as New Zealand, United Kingdom, some African countries and parts of Australia, United States of America and Europe (36). However, if BioscoreDairy is used in such countries the weightings of scores may be different due to different expert opinions and the epidemiology of infectious bovine diseases. For example, in many parts of New Zealand calvings occur outdoors (37) in comparison to Ireland where most farms carry out calving indoors (38). There is also potential to develop a BioscoreBeef specifically for pasture-based beef enterprises as they operate in Ireland.

There are some limitations to our study. The BWS resulted in some weightings which “mis-ordered” the question responses. During review, mis-ordered responses were corrected by the project team, by either averaging across responses, or by making the question score neutral. However, this approach is unlikely to have resulted in the ‘correct’ allocation of weightings for these specific questions. It is unclear why misordering of response weightings may have occurred but may have been a misunderstanding of the statements by some experts for specific questions. Our intended approach to mitigate this risk was to conduct scoring during in an interactive webinar in which experts were free to ask questions where confusion arose. However, this effect may still have persisted for some questions. Another limitation of this study was the use of an expert opinion approach to scoring. While this is a standard approach in development of such biosecurity scoring audits (33) and trans-disciplinary expertise was enrolled from multiple specialist sources, nevertheless it is a subjective process, though one which also has the advantages of harnessing stakeholders with deep sectoral knowledge of this specialised topic.

Whilst our study makes use of existing databases to ensure robust/objective inputs where possible (e.g., cattle introductions), the questionnaire aspect of the tool relies, like other systems, on farmer responses. There are several reasons why these responses therefore may be an inaccurate reflection of management on the farm. Firstly, there may be a mismatch between what the farmer perceives to be occurring on the farm, compared with actual practices implemented on the farm, the issue of farm-blindness (34); secondly, farmer responses may indicate practice at a particular point in time which may not be reflective of practice if measured over a longer time period. Finally, in many cases, farmers are likely to be aware that the practice implemented on their farm does not conform to best practice, and therefore may give responses which do not reflect their true management practices. In other contexts, the term social desirability bias is often used to describe this effect (35), a recognised approach advocated for addressing this type of bias includes providing the respondent with assurances regarding anonymity and confidentiality. The approach taken with our study, in developing a system that is not delivered by an animal health professional may mitigate this impact since there is no interviewer present at data collection. In addition, like most data collections it is important that farmers are reassured regarding where the data goes, what is it used for and who will be handling the data or personal information. In order to reassure farmers, all data should be anonymised by allocating a response number to their completed survey, and stored in a secure file. Farmers should be provided with a detailed description of how their data would be managed and assessed.

## 5 Conclusion

Multiple biosecurity risk assessment tools have been developed to audit cattle farms nationally and transnationally, but none were deemed suitable for pasture-based dairy enterprises as they operate in Ireland. The tool developed here, BioscoreDairy, is unique in combining both questionnaire responses and recorded cattle movement data. The co-design methodology adopted in producing the questionnaire and in applying the best-worst scaling method also constituted a novel approach to assigning score weightings across a broad range of experts during the design of a farmer-facing biosecurity risk assessment tool.

## Data Availability

The datasets presented in this study can be found in online repositories. The names of the repository/repositories and accession number(s) can be found in the article/Supplementary material.

## Footnotes

^1^ https://animalhealthireland.ie/about/who-we-are/technical-working-groups/biosecurity-technical-working-group/

## References

[1] WOAH. For the purposes of the [WWW document]. World Organ. Anim. Heal. (2022). Available at: https://www.woah.org/fileadmin/Home/eng/Health_standards/tahc/current/glossaire.pdf (Accessed November 5, 2024)

[2] Kennedy AE O’Doherty EF Byrne N O’Mahony J Kennedy EM Sayers RG. A survey of management practices on Irish dairy farms with emphasis on risk factors for Johne’s disease transmission. Ir Vet J. (2014) 67:27–11. doi: 10.1186/s13620-014-0027-9, PMID: 25610611 PMC4300563

[3] More SJ McKenzie K O’Flaherty J Doherty ML Cromie AR Magan MJ. Setting priorities for non-regulatory animal health in Ireland: results from an expert policy Delphi study and a farmer priority identification survey. Prev Vet Med. (2010) 95:198–207. doi: 10.1016/j.prevetmed.2010.04.011 20554068

[4] Mallioris P Teunis G Lagerweij G Joosten P Dewulf J Wagenaar JA. Biosecurity and antimicrobial use in broiler farms across nine European countries: toward identifying farm-specific options for reducing antimicrobial usage. Epidemiol Infect. (2023) 151:e13. doi: 10.1017/S0950268822001960, PMID: 36573356 PMC9990406

[5] Brennan ML Christley RM. Biosecurity on cattle farms: a study in north-west England. PLoS One. (2012) 7:e28139. doi: 10.1371/journal.pone.0028139, PMID: 22235244 PMC3250388

[6] Sumner CL von Keyserlingk MAG Weary DM. How benchmarking motivates farmers to improve dairy calf management. J Dairy Sci. (2018) 101:3323–33. doi: 10.3168/jds.2017-13596, PMID: 29397181

[7] biocheck.Ghent. Biocheck Pigs [WWW document]. (2023). Available at: https://biocheckgent.com/sites/default/files/2023-03/Pig_EN_V3.0.pdf (Accessed November 5, 2024)

[8] biocheck.Ghent. Biocheck poultry [WWW document]. (2023). Available at: https://biocheckgent.com/sites/default/files/2023-02/Layer_2.0_EN.pdf (Accessed November 5, 2024)

[9] biocheck.Ghent. Biocheck-Dairy [WWW document]. (2023). Available at: https://biocheckgent.com/sites/default/files/2023-03/Dairy_EN_V2.0.pdf (Accessed November 5, 2024)

[10] biocheck.Ghent. Biocheck-Beef [WWW document]. (2023). Available at: https://biocheckgent.com/en/questionnaires/beef-cattle (Accessed November 5, 2024)

[11] McCarthy MC O’Grady L McAloon CG Mee JF. A survey of biosecurity and health management practices on Irish dairy farms engaged in contract-rearing. J Dairy Sci. (2021) 104:12859–70. doi: 10.3168/jds.2021-20500 34593236

[12] Mee JF Geraghty T O’Neill R More SJ. Bioexclusion of diseases from dairy and beef farms: risks of introducing infectious agents and risk reduction strategies. Vet J. (2012) 194:143–50. doi: 10.1016/j.tvjl.2012.07.001 23103219 PMC7110757

[13] Choi BCK Pak AWP. A catalog of biases in questionnaires. Prev Chronic Dis. (2005) 2:1–13. PMC1323316 15670466

[14] Kelly P Shalloo L Wallace M Dillon P. The Irish dairy industry – recent history and strategy, current state and future challenges. Int J Dairy Technol. (2020) 73:309–23. doi: 10.1111/1471-0307.12682

[15] Mee JF Barrett D Boloña PS Conneely M Earley B Fagan S. Ruminant health research – progress to date and future prospects, with an emphasis on Irish research. Irish J Agric Food Res. (2022) 61:55–86. doi: 10.15212/ijafr-2020-0150

[16] Osawe OW Läpple D Mee JF. Economic analysis of biosecurity adoption in dairy farming: evidence from Ireland. J Anim Sci. (2022) 100:skac218. doi: 10.1093/jas/skac218, PMID: 35700524 PMC9492279

[17] Department of Agriculture, Food and the Marine. NFABS 2021-2024. A National Farmed Animal Biosecurity Strategy 1, 1–44. (2021). Available at: https://www.gov.ie/en/publication/d8cbf-animal-health-welfare-biosecurity/ (Accessed November 5, 2024)

[18] Damiaans B Renault V Sarrazin S Berge AC Pardon B Saegerman C. A risk-based scoring system to quantify biosecurity in cattle production. Prev Vet Med. (2020) 179:104992. doi: 10.1016/j.prevetmed.2020.104992, PMID: 32438203

[19] McAloon CG Doherty ML Whyte P More SJ O’Grady L Citer L. Relative importance of herd-level risk factors for probability of infection with paratuberculosis in Irish dairy herds. J Dairy Sci. (2017) 100:9245–57. doi: 10.3168/jds.2017-12985 28888596

[20] Department of Agriculture, Food and the Marine. AIM [WWW document]. (2020). Available at: https://www.gov.ie/en/publication/68686-animal-identification-movement-aim/ (Accessed November 5, 2024)

[21] Meadows K. Cognitive interviewing methodologies. Clin Nurs Res. (2021) 30:375–9. doi: 10.1177/10547738211014099 33998325

[22] Willis GB Artino AR Jr. What do our respondents think We’re asking? Using cognitive interviewing to improve medical education surveys. J Grad Med Educ. (2013) 5:353–6. doi: 10.4300/JGME-D-13-00154.1, PMID: 24404294 PMC3771159

[23] SurveyMonkey. Survey monkey [WWW document]. Surv. Quest. (2023). Available at: https://www.surveymonkey.com/welcome/sem/?program=7013A000000mweBQAQ&utm_bu=CR&utm_campaign=71700000059184849&utm_adgroup=58700005410221933&utm_content=43700049188946195&utm_medium=cpc&utm_source=adwords&utm_term=p49188946195&utm_kxconfid=s4bvpi0ju&langua (Accessed November 5, 2024)

[24] Louviere JJ Flynn TN Marley AAJ. Best-worst scaling: theory, methods and applications In: Louviere JJ, Flynn TN, Marley AAJ, (ediotrs). Best-worst scaling theory, methods appl. UK: Cambridge University Press (2015). 1–342.

[25] Tsafarakis S Gkorezis P Nalmpantis D Genitsaris E Andronikidis A Altsitsiadis E. Investigating the preferences of individuals on public transport innovations using the maximum difference scaling method. Eur Transp Res Rev. (2019) 11:1–12. doi: 10.1186/s12544-018-0340-6

[26] Conjointly. Conjointly [WWW document]. Adv. Surv. Platf. with Expert Support. (2016). Available at: https://conjointly.com/ (Accessed Feburary 20, 2024).

[27] Brock J Lange M Tratalos JA More SJ Graham DA Guelbenzu-Gonzalo M. Combining expert knowledge and machine-learning to classify herd types in livestock systems. Sci Rep. (2021) 11:2989. doi: 10.1038/s41598-021-82373-3, PMID: 33542295 PMC7862359

[28] R Core Team. R: a language and environment for statistical computing. Vienna, Austria: R Foundation for Statistical Computing (2023).

[29] Richens IF Houdmont J Wapenaar W Shortall O Kaler J O’Connor H. Application of multiple behaviour change models to identify determinants of farmers’ biosecurity attitudes and behaviours. Prev Vet Med. (2018) 155:61–74. doi: 10.1016/j.prevetmed.2018.04.010, PMID: 29786526

[30] Garry F. Control of paratuberculosis in dairy herds. Vet Clin Food Anim Pract. (2011) 27:599–607. doi: 10.1016/j.cvfa.2011.07.006 22023838

[31] Geraghty T Graham DA Mullowney P More SJ. A review of bovine Johne’s disease control activities in 6 endemically infected countries. Prev Vet Med. (2014) 116:1–11. doi: 10.1016/j.prevetmed.2014.06.003, PMID: 24997766

[32] McAloon CG Macken-Walsh Á Moran L Whyte P More SJ O’Grady L. Johne’s disease in the eyes of Irish cattle farmers: a qualitative narrative research approach to understanding implications for disease management. Prev Vet Med. (2017) 141:7–13. doi: 10.1016/j.prevetmed.2017.04.001, PMID: 28532994

[33] Amalraj A Van Meirhaeghe H Caekebeke N Creve R Dufay-Lefort A-C Rousset N. Development and use of Biocheck.UGent™ scoring system to quantify biosecurity in conventional indoor (Turkey, duck, breeder) and free-range (layer and broiler) poultry farms. Prev Vet Med. (2024) 230:106288. doi: 10.1016/j.prevetmed.2024.106288, PMID: 39067265

[34] Mee JF. Denormalizing poor dairy youngstock management: dealing with “farm-blindness”. J Anim Sci. (2020) 98:S140–9. doi: 10.1093/jas/skaa137, PMID: 32810251 PMC7433914

[35] Larson RB. Controlling social desirability bias. Int J Mark Res. (2019) 61:534–47. doi: 10.1177/1470785318805305

[36] Joubran AM Pierce K Garvey N Shalloo L O’Callaghan T. Invited review: A 2020 perspective on pasture-based dairy systems and products. J Dairy Sci. (2021) 104:7364–382. 10.3168/jds.2020-19776 33865573

[37] Neave HW Sumner C Henwood R Zobel G Saunders K Thoday H. Dairy farmers’ perspectives on providing cow-calf contact in the pasture-based systems of New Zealand. J Dairy Sci. (2022) 105:453–67, PMID: 34696913 10.3168/jds.2021-21047

[38] Crossley RE Bokkers EAM Browne N Sugrue K Kennedy E Conneely M. Risk factors associated with indicators of dairy cow welfare during the housing period in Irish, spring-calving hybrid pasture-based systems. Prev Vet Med. (2022) 208:105760. 10.1016/j.prevetmed.2022.105760 36181750

