# Supplementary material for: Use of conjoint analysis to weight biosecurity practices on pasture-based dairy farms to develop a novel audit tool—BioscoreDairy
Source: Front Vet Sci. 2024 Dec 10;11:1462783. doi: 10.3389/fvets.2024.1462783 (PMC11669396; doi:10.3389/fvets.2024.1462783)
Supplement: Supplementary file 1 [file Supplementary_file_1.zip › Supplementary Material and Appendix 1/Appendix 1, BioscoreDairy Questionnaire.pdf]

Welcome to the Irish Biosecurity Risk Assessment Tool.

- **This survey will only take approx. 12minutes to fill out.**
- **The survey is based on your own farm practices over the past 3 to 5 years.**
- **Questions with a * beside them require and answer**

All data collected will be anonymised and your personal information will be protected under the EU General Data Protection (EU Regulation 2016/679).
Data will be stored securely and destroyed upon completion of the trial.Your data will not be shared with any third parties for any reason.This study has ethical exemption from UCD's HREC-LS committee.

Thank you for participating in our survey. Your feedback is important.

* 1. Do you consent to taking part in this questionnaire?

◯ Yes

◯ No

Introduction

This research is focusing on Infectious diseases on Irish dairy herds. By completing this survey we hope to have a clear understanding of the biosecurity status among Irish dairy farms. Therefore acknowledge areas to improve biosecurity on farm as addressed in a new Animal Health Law (2021)

Key Aims:
1. To help identify risks of disease entry and factors contributing to spread of infectious disease among our herds
2. To investigate methods of reducing these risks

Your data will be anonymised and only research members will have access to this information.

* 1. Do you use a contract rearing service?

- Yes
- No

Section 1 – Risk of disease entry to your herd

This section assesses the risk of a significant infection entering your herd

* 1. When purchasing animals, where do you primarily buy them from?

(Please select **only one** option)

- ☐ Mart
- ☐ Auction/Show and Sale
- ☐ Private source
- ☐ I do not purchase animals

1. For animals that you purchased in the past, how were they transported to your farm (select any that have applied in the past 5 years)

	Yes	No
Haulier	○	○
Own Trailer	○	○
Borrowed Trailer	○	○

2. In any of these cases, were other animals (from, or destined for a different farm) transported in the same trailer at the same time?

- ○ Always
- ○ Sometimes
- ○ Never

3. In the past 3 years, when you have bought in animals, did you enquire about the status of the source herd for any of the following diseases

	Yes	No
TB	○	○
BVD	○	○
Johne’s Disease	○	○
Mycoplasma bovis	○	○
Salmonella	○	○
IBR	○	○
Mortellaro/ digital dermatitis	○	○
Campylobacter	○	○

4. For cattle that you have purchased in the past 3 years, How often were these cattle tested?

	Never	Sometimes	Always
IBR	○	○	○
Johne's Disease	○	○	○
Leptospirosis	○	○	○
Mycoplasma bovis	○	○	○
Neospora	○	○	○
Salmonella	○	○	○
Campylobacter	○	○	○

5. Where cattle tested positive for the above diseases, how often were these cattle excluded/culled?

	Never	Sometimes	Always	N/A
IBR	○	○	○	○
Johne’s disease	○	○	○	○
Leptospirosis	○	○	○	○
Mycoplasma bovis	○	○	○	○
Neospora	○	○	○	○
Salmonella	○	○	○	○
Campylobacter	○	○	○	○

6. When you have purchased cattle in the past, did you place them in quarantine* as soon as they arrived on the farm

*Quarantine is isolating bought animals in an area physically separated from other animals (this includes not sharing airspace with any other animals).

- ○ Always
- ○ Sometimes
- ○ Never

1. If you have quarantined cattle in the past, what is the **minimum** time you quarantined these cattle on your farm for?

- ◯ Less than 7 days
- ◯ 8 to 27 days
- ◯ 28 days or longer

2. Are purchased cattle given the following treatments upon arrival or during quarantine period on the farm?

	Never	Sometimes	Always
Vaccination	◯	◯	◯
Foot Bath	◯	◯	◯
Worm Treatment	◯	◯	◯
Lice/Mange Treatment	◯	◯	◯
Fluke Treatment	◯	◯	◯

3. When you have purchased milking cows in the past 3 years, have you milked them separately during the quarantine period?

- ◯ Yes, they are milked first
- ◯ Yes, they are milked last
- ◯ Not Applicable- Do not purchase milking cows
- ◯ No

4. Over the past 3 years, have any cattle been moved off and then returned to the farm for any of the following reasons?

	Yes	No
Livestock show	◯	◯
Contract Rearing	◯	◯
Unsold Animals from the Mart	◯	◯
Hiring/Loan in/out of bulls	◯	◯

Other (please specify)

[ ]

* 1. Were cattle who left your herd and returned for any of the reasons stated in the previous question, quarantined on return to your farm?

- Yes
- No
- Not Applicable

* 1. From the list below, please indicate whether disinfection measures were used upon visiting your farm within the past 12months

	Measure
Vet	
Hoof Trimmer	
Scanner	
Dead stock collector	
Discussion Group	
AI Technician	

* 2. In the past 3 years, has slurry from <u>other</u> cattle **or** pig farms been spread on your pasture?

- Yes
- No

* 3. In the past 3 years, have your cattle had access to water from either a river, stream, pond or lake?

- Cattle **have** access to water courses (streams, rivers, ponds lakes)
- Cattle **sometimes** have access to water courses (streams, rivers, ponds lakes)
- Cattle **do not have** access to water courses (streams, rivers, ponds lakes)

* 4. Have you grazed any of your cattle on rented ground which may have been grazed by other stock or had slurry/dung applied in the 12 months prior to your cattle grazing?

- Yes
- No

* 5. Have you rented out any of your ground for other animals to graze (e.g. wintering sheep) and then had your own animals graze that land again within 12 months of other animals leaving?

- Yes
- No

* 6. Do you use a contractor to spread slurry on your farm?

- Always
- Sometimes
- Never

1. Contractors used to spread slurry- If yes (sometimes/always), has the equipment been washed out and cleaned before it enters your farm?

- Yes
- No
- I don't know

* 2. In the past 3 years, have you used colostrum or milk from another farm to feed any of your calves?

- Yes
- No

* 3. Do you, or any of your farm workers (including family workers) also work or frequently visit (i.e. at least once per week) any other farms?

- Yes
- No
- Don't Know

4. Do these workers use separate overalls/boots for visiting/working at the second farm?

- Yes
- No

* 5. Is it possible for your cattle to make nose to nose contact with other animals from neighbouring farms when grazing?

- Yes
- No, there are animals on a neighbouring farm but there is a physical boundary around the boundary (river, drain, double fenced)
- No, there are no animals from a neighbouring farm grazing at the boundaries (arable land/forestry)

1. What animals are your cattle exposed to nose to nose contact with on neighbour land ?

- [ ] Cattle
- [ ] Sheep
- [ ] Goats
- [ ] Alpaca
- [ ] Llama
- [ ] Other (please specify)

* 2. In the past 3 years, have you used handling facilities that are also shared with another herd?

- ( ) Yes
- ( ) No

* 3. Have you seen evidence of deer on your land?

- ( ) Yes
- ( ) No

* 4. Have you seen evidence of badgers on your land (e.g. sighting, setts, dung, signs of activity)

- ( ) Yes
- ( ) No

* 5. When at pasture, are your feed troughs and water drinkers >1m above the level of the ground?

- ( ) All
- ( ) Some
- ( ) None

Section 2. Speed of disease spread within your herd

This section assesses the speed at which a significant infection might spread within your herd

* 1. Do you have a specific pen (which is not used for any other purpose e.g. calving) in which you can fully isolate sick/lame/aborted/Johne's Disease positive or TB reactor animals?

- ○ Yes, but this is in the same airspace as other animals
- ○ Yes, and animal is in separate airspace to other animals
- ○ No

* 2. Are sick animals always kept in this pen?

- ○ Always
- ○ Most of the time
- ○ Sometimes
- ○ Occasionally
- ○ Never
- ○ N/A

* 3. Are sick cattle taken care of (fed/watered) before or after healthy cattle or calves?

- ○ Before
- ○ After
- ○ No specific order

* 4. Is animal feed (i.e. concentrates) stored in a secure area away from pests/rodents/other animal feed?

- ○ Yes
- ○ No

* 5. Do any of the following age groups share airspace with older animals at housing? (note: colour coding in place to differentiate age groups- therefore **please only select one of each coloured statement- Black, pink, green, and blue**)

- ☐ **Pre-weaned calves have shared airspace with heifers**
- ☐ **Pre-weaned calves have no shared airspace with heifers**
- ☐ **Pre-weaned calves have contact with adult animals**
- ☐ **Pre-weaned calves have no contact with adult animals**
- ☐ **Weaned calves/maiden heifers have contact with adult animals**
- ☐ **Weaned calves/maiden heifers have no contact with adult animals**
- ☐ **In calf heifers have contact with adult animals**
- ☐ **In calf heifers have no contact with adult animals**

* 6. Where do the majority of heifers/cows calve?

- Group calving pen (with/without individual pens)
- Individual calving pens only
- Outdoor pad/paddock

* 7. Are calving pens ever used to house sick/lame/aborted/positive Johne's disease positive animals/TB reactors?

- Sometimes
- Never

* 8. What is the cleaning procedure for your calving facilities at each of the following time points?

	After Every Calving	After Multiple Calvings	Between calving season's	Not Standard Practice
Partial clean out (remove dirty bedding)	○	○	○	○
Complete clean out (remove all bedding)	○	○	○	○
Wash/ Power hose/ steam clean	○	○	○	○
Disinfection of floor / walls after complete clean out and wash	○	○	○	○

* 9. When does the separation of the calf from the dam most commonly take place? Select one option.

- Most calves are removed from the dam within 1 hour of being born
- Most calves spend more than 1 hour but less than 12 hours with the dam, (e.g. Calves are routinely left with the cow and moved at the next milking)
- Most calves spend more than 12 hours but less than 3 days with the dam
- Most calves spend more than 3 days with the dam

* 10. If an animal aborts on the farm, where are the foetal membranes and tissues (placenta/cleaning) disposed of from this animal?

- They are left in the calving pen
- Removed immediately from calving pen
- They are put in the manure pile or slurry pit

Other (please specify)

[ ]

* 11. Are pre-weaned calves ever housed singly in individual pens or individual hutches?

- For less than 1 week
- For the entire pre-weaning period
- Never

* 12. What is the maximum number of calves allocated to any pre-weaning pen?

* 13. Do you ever hold older calves back with younger calves depending on size/weight gain/ or any other reason?

- Routinely
- Sometimes
- Never

* 14. What is the cleaning procedure for housing facilities used for pre-weaned calves?

	After each batch of calves	After multiple batches of calves	Between Seasons	Not Standard Practice
Partial clean out (remove dirty bedding)				
Complete clean out (remove all bedding)				
Wash, Power hose, steam clean				
Disinfection of floor / walls after complete clean out and wash				

* 15. What is the shortest interval (from application) that you have grazed animals on grass that has had slurry applied to it?

Please specify between days/weeks/months etc

* 16. Aside from cattle, do you have any of the following animals on your farm, and do these animals have contact with any of your cattle, either at pasture or while being housed?

	Present
Sheep/ Goats	
Dog	
Alpaca/ Llama	

Section 2. Speed of disease spread within your herd

This section assesses the speed at which a significant infection might spread within your herd

* 1. What is the source of colostrum for most of your calves?

	Colostrum Source
Replacement Calves	
Non- Replacement Calves	

* 2. Do calves always receive colostrum pasteurised at 60°C for 60 minutes

- ○ Replacement Calves
- ○ Non-Replacement Calves
- ○ No, Colostrum is not pasteurised

* 3. How are most of your calves fed colostrum?

Replacement Calves	
Non-Replacement Calves	

* 4. How much colostrum (in litres) is typically fed within the first 6 hours of life?

* 5. How often is waste (non-saleable) milk fed to calves?

- ○ Routinely
- ○ Occasionally
- ○ Never

Section 3 – Diagnosis of infection

This section assesses the speed at which you are likely to detect a significant infection in your herd.

* 1. Do you Milk Record ?

☐ Yes ☐ No

* 1. Do you review milk recording results regularly with your vet/team?

- ◯ Yes
- ◯ No

* 2. Are Bulk Milk Tank disease tests, individual animal milk disease tests or regular herd blood tests (from healthy animals) carried out?

◯ Yes ◯ No

1. If yes for which disease(s) ?

	Bulk Tank	Individual	Routine Blood Test
Johnes ’s disease	◯	◯	◯
IBR	◯	◯	◯
Mycoplasma	◯	◯	◯
Salmonella	◯	◯	◯
Neospora	◯	◯	◯
Leptospirosis	◯	◯	◯

2. Do you use factory/Abatoir reports to routinely monitor fluke and pneumonia rates?

◯ Yes ◯ No

1. Do you discuss these results with your vet and/or farm team?

- ◯ Yes
- ◯ No

2. Do you routinely record all clinical disease problems on farm?

◯ Yes ◯ No

1. Do you discuss these results regularly with your vet and/or farm team?

- ◯ Yes
- ◯ No

2. Do you record all of the following on your farm?

	Yes	No
Calf scour	◯	◯
Respiratory (pneumonia)	◯	◯
Abortion	◯	◯
Lameness	◯	◯
Mastitis	◯	◯

3. Do you review all cases of the following with your vet?

	Yes	No
Calf scour	◯	◯
Respiratory (pneumonia)	◯	◯
Abortion	◯	◯
Lameness	◯	◯
Mastitis	◯	◯

4. How often are clinical disease outbreaks investigated (samples submitted to a lab)?

	Frequently	Sometimes	Rarely	Never
Calf scour	◯	◯	◯	◯
Respiratory (pneumonia)	◯	◯	◯	◯
Abortion	◯	◯	◯	◯
Lameness	◯	◯	◯	◯
Mastitis	◯	◯	◯	◯

5. How often are animals that die on farm submitted for a postmortem exam?

- ◯ Frequently
- ◯ Sometimes
- ◯ Rarely
- ◯ Never

Section 4- Vaccination/ Baseline Resilience

This section assesses your herds' likely resilience to a significant infection that enters and spread between animals

1. Which of the following do you vaccinate for, and which age groups are given the vaccine?

	0-3 Month Calves	3-12 Month Animals	12-24 Month Animals	Cows	Stock Bull	I do Not Vaccinate
Scour	☐	☐	☐	☐	☐	☐
IBR	☐	☐	☐	☐	☐	☐
Other respiratory vaccines (e.g. RSV/PI3/histophilus /Pasteurella	☐	☐	☐	☐	☐	☐
Clostridial diseases (e.g. blackleg)	☐	☐	☐	☐	☐	☐
Salmonella	☐	☐	☐	☐	☐	☐
BVD	☐	☐	☐	☐	☐	☐
Leptospirosis	☐	☐	☐	☐	☐	☐
Lungworm	☐	☐	☐	☐	☐	☐
Other e.g. Autogenous/ imported	☐	☐	☐	☐	☐	☐

2. Are Worming products administered? (Choose those that apply)

	Heifers	Lactating Cows
At particular times of the grazing season (e.g. 3 or 5 weeks post turn out, middle of the summer etc.)	☐	☐
Only after a monitoring test (e.g. faecal egg count from the group indicates treatment is required)	☐	☐
Only after weighing the group demonstrates a reduction in expected average daily gain	☐	☐
Based on level of antibodies in the bulk tank	☐	☐
Only after the appearance of clinical signs (e.g. coughing or scouring at grass)	☐	☐

3. Are Silage/Forage analysis carried out?
(place an X in the box(s) that apply)

	Always	Sometimes	Never
Full Analysis- Including minerals	○	○	○
Partial Analysis	○	○	○

* 1. Does a suitable qualified nutritionist create and regularly review the diets being fed?

◯ Yes ◯ No

1. How often does a nutritionist visit the farm to review diet and feeding?

Please specify if your answer is in Months/yearly etc

* 2. Are body condition scores monitored regularly and results discussed with farm team/ nutritionist/ vet?

  - Yes
  - No

* 3. Are locomotion scores monitored regularly and results discussed with farm team/ vet?

  - Yes
  - No

* 4. Are EBI scores used to help select breeding animals (semen/embryo’s/ breeding stock)?

  - Yes
  - No

* 5. Are health traits within EBI scores part of the selection criteria?

  - Yes
  - No

* 1. Please enter your herd number

Thank you for participating. We really appreciate you taking the time to complete our survey.
