# Supplementary material for: Use of conjoint analysis to weight biosecurity practices on pasture-based dairy farms to develop a novel audit tool—BioscoreDairy
Source: Front Vet Sci. 2024 Dec 10;11:1462783. doi: 10.3389/fvets.2024.1462783 (PMC11669396; doi:10.3389/fvets.2024.1462783)
Supplement: Supplementary file 1 [file Supplementary_file_1.zip › Supplementary Material and Appendix 1/Supplementary Material 1 - Weightings Tables S1, S2, S3.docx]

**Table S1** Weightings of practices which affect risk of disease introduction into a herd

| **Question** | **Level** | **Weighting** |
| --- | --- | --- |
| 1 | No cattle purchased | 0.546 |
| 1 | Only purchase cattle direct from individual farms | 0.212 |
| 1 | Purchasing cattle from mart | -0.260 |
| 2 | Animals moved onto farm using an owned trailer | 0.112 |
| 2 | Animals moved onto farm using a haulier | -0.126 |
| 2 | Animals moved onto farm using a borrowed trailer | -0.160 |
| 3 | Cattle moving onto farm are never transported with animals from other farms | 0.448 |
| 3 | Cattle moving onto farm are sometimes transported with animals from other farms | -0.332 |
| 3 | Cattle moving onto farm are always transported with animals from other farms | -0.356 |
| 4 | The health status of source herds for purchased animals is always known | 0.453 |
| 4 | The health status of source herds for purchased animals is sometimes known | 0.149 |
| 4 | The health status of source herds for purchased animals is never known | -0.454 |
| 5 | Purchased animals are always tested prior to arrival on farm | 0.333 |
| 5 | Animals that test positive are always not purchased | 0.206 |
| 5 | Purchased animals are sometimes tested prior to arrival on farm | 0.070 |
| 5 | Animals that test positive are still purchased | -0.250 |
| 5 | Purchased animals are not tested prior to arrival on farm | -0.295 |
| 6 | Purchased animals always quarantined before entering main herd | 0.396 |
| 6 | Purchased animals sometimes quarantined before entering main herd | 0.149 |
| 6 | Purchased animals are not quarantined before entering main herd | -0.343 |
| 7 | Minimum quarantined period over 28 days | 0.386 |
| 7 | Minimum quarantined period 8-27 days | 0.026 |
| 7 | Minimum quarantined period less than 7 days | -0.111 |
| 8 | Purchased animals are always vaccinated | 0.146 |
| 8 | Purchased animals are sometimes vaccinated | 0.127 |
| 8 | Purchased animals are always foot bathed | 0.114 |
| 8 | Purchased animals are always worm treated | 0.058 |
| 8 | Purchased animals are sometimes Fluke treated | 0.000 |
| 8 | Purchased animals are never lice/mange treated | 0.000 |
| 8 | Purchased animals are sometimes foot bathed | -0.076 |
| 8 | Purchased animals are sometimes worm treated | -0.082 |
| 8 | Purchased animals are always lice/mange treated | 0.000 |
| 8 | Purchased animals are never Fluke treated | 0.000 |
| 8 | Purchased animals are sometimes lice/mange treated | 0.000 |
| 8 | Purchased animals are never foot bathed | -0.129 |
| 8 | Purchased animals are always Fluke treated | 0.000 |
| 8 | Purchased animals are never worm treated | -0.180 |
| 8 | Purchased animals are never vaccinated | -0.188 |
| 9 | Quarantined milking cows are milked last after the rest of the herd | 0.171 |
| 9 | Quarantined milking cows are milked before the rest of the herd | -0.089 |
| 9 | Quarantined milking cows are not milked separately to the rest of the herd | -0.173 |
| 10 | Animals moved off the farm for show/unsold at mart/hired bull are not returned to herd | 0.220 |
| 10 | Animals moved off the farm for show/unsold at mart/hired bull ARE returned to herd | -0.292 |
| 11 | Animals that leave and return to the farm are always quarantined | 0.230 |
| 11 | Animals that leave and return to the farm are sometimes quarantined | -0.020 |
| 11 | Animals that leave and return to the farm are never quarantined | -0.193 |
| 12 | Vets visiting the farm disinfected overalls and/or boots on arrival. | 0.315 |
| 12 | Dead stock collectors visiting the farm disinfected overalls and/or boots on arrival | 0.256 |
| 12 | AI Technicians visiting the farm disinfected overalls and/or boots on arrival | 0.123 |
| 12 | AI Technicians don’t visit the farm | 0.114 |
| 12 | Discussion groups visiting the farm disinfected overalls and/or boots on arrival | 0.113 |
| 12 | Hoof trimmers visiting the farm disinfected overalls and/or boots on arrival | 0.093 |
| 12 | Scanners don’t visit the farm | 0.083 |
| 12 | Scanners visiting the farm disinfected overalls and/or boots on arrival | 0.025 |
| 12 | Dead stock collectors don’t visit the farm | -0.024 |
| 12 | Discussion groups visiting the farm use no disinfection measures | -0.073 |
| 12 | Hoof trimmers don’t visit the farm | -0.074 |
| 12 | Scanners visiting the farm use no disinfection measures | -0.092 |
| 12 | Vets don’t visit the farm. | -0.114 |
| 12 | Discussion groups don’t visit the farm | -0.115 |
| 12 | Dead stock collectors visiting the farm use no disinfection measures | -0.181 |
| 12 | Hoof trimmers visiting the farm use no disinfection measures | -0.199 |
| 12 | AI Technicians visiting the farm use no disinfection measures | -0.237 |
| 12 | Vets visiting the farm use no disinfection measures | -0.317 |
| 13 | Cattle/pig slurry is not imported | 0.229 |
| 13 | Cattle/pig slurry is imported | -0.259 |
| 14 | Cattle do not have access to water courses (streams, rivers, ponds lakes) | 0.117 |
| 14 | Cattle sometimes have access to water courses (streams, rivers, ponds lakes) | -0.046 |
| 14 | Cattle have access to water courses (streams, rivers, ponds lakes) | -0.122 |
| 15 | Cattle not allowed to graze land which had dung or slurry from other cattle applied in the last 12 months | 0.152 |
| 15 | Cattle allowed to graze on land which was grazedor slurry by other stock in the last 12 months | -0.151 |
| 17 | Contractor never used to spread slurry | 0.108 |
| 17 | Contractor always used to spread slurry | -0.144 |
| 17 | Contractor sometimes used to spread slurry | -0.144 |
| 18 | Contractor equipment always washed and cleaned out prior to entry on to farm | 0.200 |
| 18 | Contractor equipment is not (or unsure if) washed and cleaned out prior to entry on to farm | -0.157 |
| 19 | Colostrum or milk from other farms not used | 0.352 |
| 19 | Colostrum or milk used from other farms | -0.201 |
| 20b | Farm labour required to use separate overalls/boots etc. when working on farm, if working on other farms | 0.321 |
| 20a | No labour/family on farm who frequently visit/work on other farms (once a week minimum) | 0.108 |
| 20a | Labour/family on farm who frequently visit/work on other farms (once a week minimum) | -0.257 |
| 21 | Cattle have no nose to nose contact with neighbouring farms (Forestry/ tillage) | 0.418 |
| 21 | Cattle have no nose to nose contact with neighbouring farms (double fencing/ river/ drain) | 0.305 |
| 21 | Cattle have nose to nose contact with neighbouring herds | -0.326 |
| 22 | Shared handling facilities not used | 0.215 |
| 22 | Shared handling facilities used | -0.167 |
| 23 | Deer cannot access the farm | 0.109 |
| 23 | Deer have been seen on the farm | -0.094 |
| 24 | Badgers cannot access the farm | 0.161 |
| 24 | Badgers have been seen on the farm | -0.022 |
| 25 | Water and feed troughs in paddocks are >1m above ground level | 0.154 |
| 25 | Water and feed troughs in paddocks are <1m above ground level | -0.045 |

**Table S2** Weightings of practices which affect infection transmission within a dairy herd

| **Question** | **Level** | **Weighting** |
| --- | --- | --- |
| 26 | No dedicated isolation pen in place | 0.000 |
| 26 | Dedicated isolation pen - same airspace as other cattle | 0.000 |
| 26 | Dedicated isolation pen - no shared airspace as other cattle | 0.000 |
| 27 | Sick animals are always kept in a dedicated isolation pen with no shared air space with other animals | 0.403 |
| 27 | Sick animals are most of the time kept in a dedicated isolation pen with shared air space with other animals | -0.157 |
| 27 | Sick animals are only sometimes kept in a dedicated isolation pen | -0.178 |
| 27 | Sick animals are never kept in dedicated isolation pen | -0.370 |
| 28 | Sick animals are attended to before other animals | 0.219 |
| 28 | Sick animals are attended to after other animals | -0.224 |
| 28 | Sick animals are attended to in no specific order to other animals | -0.305 |
| 29 | Animal Feed (concentrates) is always stored in a secure area away from pests/rodents and other animal feed | 0.054 |
| 29 | Animal Feed (concentrates) is not stored in a secure area away from pests/rodents and other animal feed | -0.253 |
| 30 | Pre-weaned calves have shared airspace with heifers | 0.242 |
| 30 | Pre-weaned calves have no shared airspace with heifers | 0.219 |
| 30 | Pre-weaned calves have contact with adult animals | 0.137 |
| 30 | Pre-weaned calves have no contact with adult animals | 0.109 |
| 30 | Weaned calves/maiden heifers have contact with adult animals | -0.154 |
| 30 | Weaned calves/maiden heifers have no contact with adult animals | -0.157 |
| 30 | In calf heifers have contact with adult animals | -0.212 |
| 30 | In calf heifers have no contact with adult animals | -0.250 |
| 31 | Heifers/cows are calved in group calving pens (with/out individual pens) | 0.345 |
| 31 | Heifers/cows are calved in individual calving pens | 0.112 |
| 31 | Heifers/cows are calved in outdoor pad/paddocks | -0.196 |
| 32 | Calving pens are sometimes used to house sick/lame/aborted or positive reactors of Johnes/Tb | 0.361 |
| 32 | Calving pens never used to house sick/lame/aborted or positive reactors of Johnes/Tb | -0.508 |
| 33 | Not standard practice to disinfect floor/walls after complete clean and wash of calving pens | 0.433 |
| 33 | Not standard practice to wash/power hose/ steam clean calving pens | 0.398 |
| 33 | Not standard practice to completely clean out calving pens | 0.297 |
| 33 | Not standard practice to partially clean out calving pens | 0.137 |
| 33 | Calving pens partially cleaned out after every calving | 0.134 |
| 33 | Calving pens partially cleaned out after multiple calving’s | 0.110 |
| 33 | Calving pens partially cleaned out between calving seasons | 0.089 |
| 33 | Calving pens completely cleaned out after every calving | 0.032 |
| 33 | Calving pens completely cleaned out after multiple calving’s | 0.012 |
| 33 | Calving pens completely cleaned out between calving seasons | -0.001 |
| 33 | Calving pens washed/power housed /steam cleaned after every calving | -0.029 |
| 33 | Calving pens washed/power housed /steam cleaned after multiple calving’s | -0.110 |
| 33 | Calving pens washed/power housed /steam cleaned between calving seasons | -0.140 |
| 33 | Calving pens disinfected after every calving | -0.163 |
| 33 | Calving pens disinfected after multiple calving’s | -0.187 |
| 33 | Calving pens disinfected between calving seasons | -0.310 |
| 34 | Most calves spend 3+ days with the dam | -0.021 |
| 34 | Most calves are removed from the dam within 1hour of being born | 0.117 |
| 34 | Most calves spend >1 hour but <12 hours with the dam, (e.g. Calves are routinely left with the cow and moved at the next milking) | 0.061 |
| 34 | Most calves spend >12 hours but < 3 days with the dam | -0.021 |
| 35 | Foetal membrane and tissue from calving/abortion disposed of by other | 0.000 |
| 35 | Foetal membrane and tissue from calving/abortion left in in calving pen | -0.031 |
| 35 | Foetal membrane and tissue from calving/abortion removed immediately from calving pen | -0.031 |
| 35 | Foetal membrane and tissue from calving/abortion disposed of in dung heap/slurry pit | -0.461 |
| 36 | Pre-weaned calves are housed singly in individual pens/hutches/housing for < 1 week | 0.289 |
| 36 | Pre-weaned calves are never housed singly in individual pens/hutches/housing | 0.055 |
| 36 | Pre-weaned calves are housed singly in individual pens/hutches/housing for the entire pre-weaning period | -0.083 |
| 38 | Older calves always held back where growth targets are not met | 0.000 |
| 38 | Older calves sometimes held back where growth targets are not met | 0.000 |
| 38 | Older calves never held back where growth targets are not met | 0.000 |
| 39a | Not standard practice to clean out pre- weaning housing | -0.163 |
| 39a | Pre-weaning housing cleaned out after each batch of calves | -0.040 |
| 39a | Pre-weaning housing cleaned out after multiple batches of calves | -0.162 |
| 39a | Pre-weaning housing cleaned out between seasons | -0.163 |
| 39b | Pre-weaning housing disinfected after each batch of calves | -0.234 |
| 39b | Pre-weaning housing disinfected after multiple batches of calves | -0.273 |
| 39b | Pre-weaning housing disinfected between seasons | -0.314 |
| 39b | Pre-weaning housing not disinfected | -0.407 |
| 44 | Cattle have potential contact with sheep/goats | 0.000 |
| 44 | Cattle have potential contact with dogs | 0.000 |
| 44 | Cattle have potential contact with Alpaca/Llama’s | -0.021 |
| 44 | Cattle have no potential contact with sheep/goats | 0.000 |
| 44 | Cattle have no potential contact with dogs | 0.000 |
| 44 | Cattle have no potential contact with Alpaca/Llama’s | 0.000 |
| 45 | Replacement calves fed colostrum from dam only | 0.164 |
| 45 | Replacement calves fed colostrum from a single cow that is usually not the dam of the calf | 0.156 |
| 45 | Replacement calves fed pooled colostrum (multiple cows) | 0.047 |
| 45 | Replacement calves fed pooled colostrum (some cows excluded from the colostrum) | 0.047 |
| 45 | Non- Replacement calves fed colostrum from dam only | -0.068 |
| 45 | Non- Replacement calves fed colostrum from a single cow that is usually not the dam of the calf | -0.092 |
| 45 | Non- Replacement calves fed pooled colostrum (multiple cows) | -0.152 |
| 45 | Non- Replacement calves fed pooled colostrum (some cows excluded from the colostrum) | -0.152 |
| 46 | Replacement / non replacement calves receive colostrum pasteurised at 60°C for 60mins | 0.259 |
| 46 | Calves do not receive pasteurised colostrum | -0.047 |
| 47 | Replacement calves left to suckle colostrum from dam | 0.102 |
| 47 | Replacement calves fed colostrum from a bottle/ bucket and teat | 0.117 |
| 47 | Replacement calves routinely stomach tubed colostrum with a stomach tube | 0.102 |
| 47 | Non- Replacement calves left to suckle colostrum from dam | -0.008 |
| 47 | Non- Replacement calves fed colostrum from a bottle/ bucket and teat | 0.023 |
| 47 | Non- Replacement calves routinely stomach tubed colostrum with a stomach tube | -0.008 |
| 49 | Calves never fed waste milk | 0.014 |
| 49 | Calves sometimes fed waste milk | -0.062 |
| 49 | Calves routinely fed waste milk | -0.139 |

**Table S3** Weightings of practices which affect ability to diagnose infections within a dairy herd

| **Question** | **Level** | **Weighting** |
| --- | --- | --- |
| 50 | Regularly reviewing of milk recording | 0.214 |
| 50 | Milk Recordings performed but not reviewed | -0.113 |
| 50 | Milk recording is not carried out | -0.222 |
| 51 | Routine Bulk Milk Tank disease testing | 0.210 |
| 51 | No routine Bulk Milk Tank disease testing | -0.203 |
| 52 | Routine Individual animal milk disease testing | 0.182 |
| 52 | No routine Individual animal milk disease testing | -0.174 |
| 53 | Routine Individual animal blood testing | 0.202 |
| 53 | No routine Individual animal blood testing | -0.194 |
| 54 | Factory reports are used to monitor fluke and pneumonia rates on farm | 0.189 |
| 54 | Factory reports are not used to monitor fluke and pneumonia rates on farm | -0.110 |
| 56 | All clinical disease problems are recorded and reviewed by farmer | 0.286 |
| 56 | All clinical disease problems are not recorded | -0.317 |
| 56 | All clinical disease problems are recorded and reviewed with vet and/or farm advisor | 0.516 |
| 57 | Clinical disease outbreaks are always investigated | 0.419 |
| 57 | Clinical disease outbreaks are sometimes investigated | 0.124 |
| 57 | Clinical disease outbreaks are rarely investigated | -0.280 |
| 57 | Clinical disease outbreaks are never investigated | -0.484 |
| 58 | Cattle that die on farm are always submitted for post-mortem | 0.297 |
| 58 | Cattle that die on farm are sometimes submitted for post-mortem | 0.031 |
| 58 | Cattle that die on farm are rarely submitted for post-mortem | -0.186 |
| 58 | Cattle that die on farm are never submitted for post-mortem | -0.388 |
