# Supplementary material for: Use of conjoint analysis to weight biosecurity practices on pasture-based dairy farms to develop a novel audit tool—BioscoreDairy
Source: Front Vet Sci. 2024 Dec 10;11:1462783. doi: 10.3389/fvets.2024.1462783 (PMC11669396; doi:10.3389/fvets.2024.1462783)
Supplement: Supplementary file 1 [file Supplementary_file_1.zip › Supplementary Material and Appendix 1/Supplementary Material 2 - Farm report.pdf]

Infectious Disease Risk Assessment Report

[redacted] Moorepark, Teagasc

December 2023

Introduction

Dear [redacted],
Thank you for completing the infectious disease control questionnaire. Your results, compared with other similar herds are given in this report. The first section of this report deals with cattle movement into your herd over the last 3 years. Cattle introduction is considered the greatest risk factor for the introduction of any infectious disease.
The second section of the report compares your questionnaire results to those from 200 similar dairy farmers. This section addresses practices in place on your farm to limit the risk of infection introduction and the speed that it can spread once in the herd.

Cattle movement

The risk associated with your cattle introductions is shown in the graphs below compared to 50 other similar herds. The position of your herd in each graph is indicated by the orange bar. The green line represents the position of the lowest-risk (10th percentile) herds, the black line indicates the position of the average herd, and the red line indicates the position of the highest-risk (90th percentile) herds.

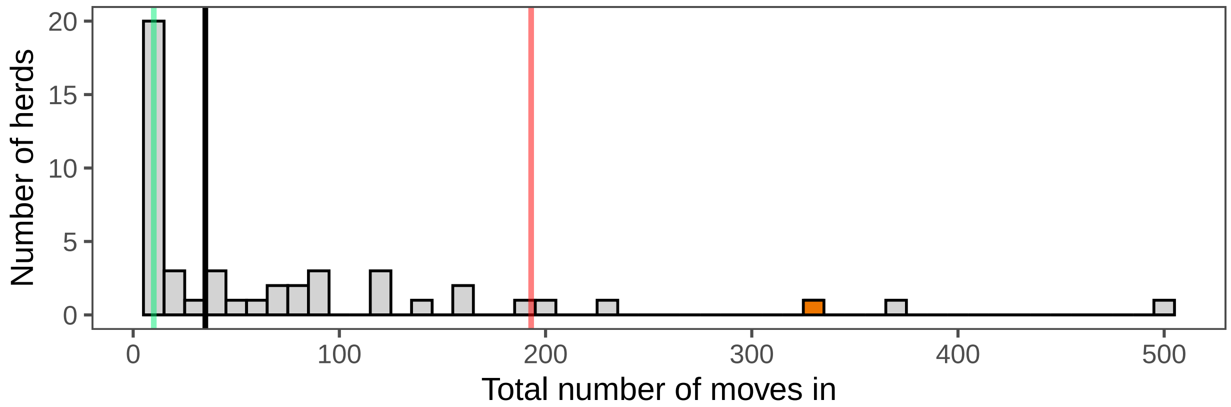

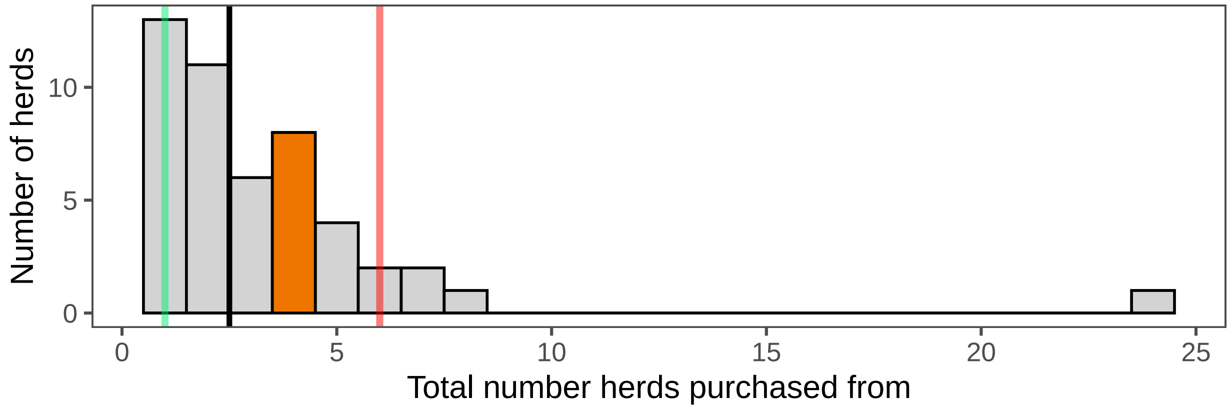

Over the last three years, there were 323 animal movements into your herd. Of these, 2 were introduced from through marts or other non-farm-to-farm sources; and overall, animals were introduced from 4 different source herds. Compared with other similar herds, yours is among the Highest-risk for total number of animals introduced; and among the Highest-risk for number of herds sourced from.

Management practices to control infectious diseases on farm

The graph below compares your questionnaire responses to 200 other dairy farms. The graph shows your score across three sections: risk of infection introduction, speed of spread, and diagnosis. Your score for each section is displayed as a percentage of the total score possible for that section and the bar is colour coded (red, orange green) according to how your farm compares to other similar dairy herds.

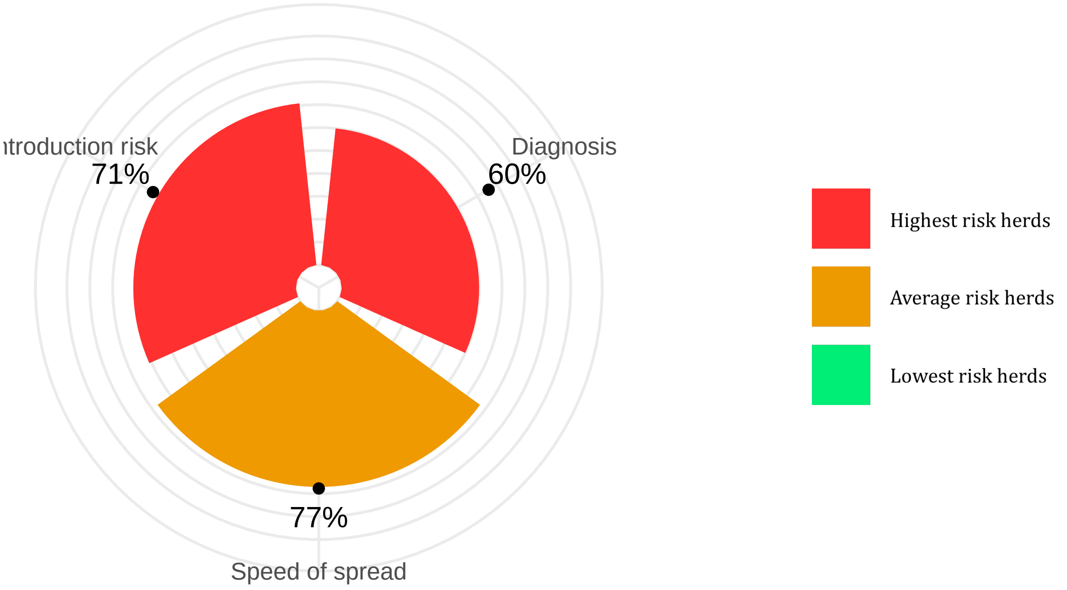

	Your herd	Average herd
Diagnosis	60	76
Introduction risk	71	74
Speed of spread	77	78

The black dot in each section represents the score of the average herd. Higher scores are better, the best possible score for each section is 100%. As can be seen from the graph, compared to other similar herds, yours is among the medium risk herds for risk of introducing infection into the herd; among the highest risk herds for controlling the speed that diseases can spread within the herd and among the highest risk herds for rapid diagnosis of infections should they be introduced to the herd.
